# Multicellular spheroids from normal and neoplastic thyroid tissues as a suitable model to test the effects of multikinase inhibitors

**DOI:** 10.18632/oncotarget.14187

**Published:** 2016-12-26

**Authors:** Valentina Cirello, Valentina Vaira, Elisa Stellaria Grassi, Valeria Vezzoli, Dario Ricca, Carla Colombo, Silvano Bosari, Leonardo Vicentini, Luca Persani, Stefano Ferrero, Laura Fugazzola

**Affiliations:** ^1^ Endocrine Unit, Fondazione IRCCS Ca’ Granda, 20122 Milan; ^2^ Department of Pathophysiology and Transplantation, University of Milan, 20122 Milan; ^3^ Division of Pathology, Fondazione IRCCS Ca’ Granda, 20122 Milan; ^4^ Laboratory of Endocrine and Metabolic Research, Istituto Auxologico Italiano IRCCS, 20149 Milan; ^5^ Department of Clinical Sciences and Community Health, University of Milan, 20122 Milan; ^6^ Endocrine Surgery Unit, Fondazione IRCCS Ca’ Granda, 20122 Milan; ^7^ Department of Biomedical, Surgical and Dental Sciences, University of Milan, 20122 Milan, Italy

**Keywords:** multicellular spheroids, thyroid cancer, multi-tyrosine kinase inhibitors, SP600125, ROCK

## Abstract

Multicellular three-dimensional (3D) spheroids represent an experimental model that is intermediate in its complexity between monolayer cultures and patients’ tumor. In the present study, we characterize multicellular spheroids from papillary (PTC) and follicular (FTC) thyroid cancers and from the corresponding normal tissues. We show that these 3D structures well recapitulate the features of the original tissues, in either the differentiated and “stem-like” components. As a second step, we were aimed to test the effects of a small multikinase inhibitor, SP600125 (SP), previously shown to efficiently induce cell death in undifferentiated thyroid cancer monolayer cultures. We demonstrate the potent effect of SP on cell growth and survival in our 3D multicellular cultures. SP exerts its main effects through direct and highly significant inhibition of the ROCK pathway, known to be involved in the regulation of cell migration and β-catenin turnover. Consistently, SP treatment resulted in a significant decrease in β-catenin levels with respect to basal conditions in tumor but not in normal spheroids, indicating that the effect is promisingly selective on tumor cells.

In conclusion, we provide the morphological and molecular characterization of thyroid normal and tumor spheroids. In this 3D model we tested *in vitro* the effects of the multikinase inhibitor SP and further characterized its mechanism of action in both normal and tumor spheroids, thus making it an ideal candidate for developing new drugs against thyroid cancer.

## INTRODUCTION

Multicellular three-dimensional spheroids represent an experimental model that is intermediate in its complexity between monolayer cultures and patients’ tumor, and are thus successfully used in cancer research [1, 2]. Differently from two-dimensional monolayer cultures, the primary spheroids retain a three-dimensional (3D) structure, forming intercellular contacts, and usually displaying radial gradients of oxygen, glucose, and other nutrients with low values in the center, thus mimicking the natural environment of solid tumors [1]. Although several kinds of cancer cells have already been cultured as multicellular spheroids and used for investigation of tumor cell growth and response to various kinds of treatments [2, 3], most *in vitro* studies on human thyroid carcinoma cells have been so far conducted on monolayer cultures [4–6].

In the last decade, novel therapeutic options have been released for radioiodine refractory thyroid cancer (TC) based on the administration of kinase inhibitors mainly directed against RAS/RAF/ERK and AKT/mTOR pathways [7]. We recently demonstrated, in normal and cancer thyroid derived cell lines, cultured in monolayer, that the kinase inhibitor SP600125 (SP) is highly effective in blocking cell growth and migration and in the induction of mitotic catastrophe through direct inhibition of ROCK, a kinase involved in the regulation of cell migration, microtubule dynamics and β-catenin turnover [8]. This mechanism of action may be particularly important in anticancer therapy considering that Rho/ROCK pathway is hyperactivated in different human neoplasia and its activity correlates with metastatic disease [9–11]. SP was found to be particularly effective against poorly differentiated cancer cells and, at a lesser extent, on papillary thyroid cancer derived cells [8].

It is well known that the *in vivo* response to antineoplastic treatments is often short lasting due to selection of resistant clones apparently originating from the cancer stem cells (CSCs) [12, 13]. Cells with stem-like properties represent a very small percentage of the thyroid cell population (<1%), but they have been detected in normal thyroid, in multinodular goiters and in thyroid cancers, though limited and controversial data exist on their characterization [14–16].

Hence, in the present study we used appropriate conditions [17] in order to maintain and possibly increase the amount of cells with stem-like properties among the heterogeneous population of the multicellular thyroid spheroids obtained from 17 papillary and 4 follicular tumor tissues, and from the matched normal tissues, and we aimed to get more insights into the effects of SP by testing it in our multicellular spheroid model which recreates in culture the typical 3D architecture of the tissue.

## RESULTS

### Thyroid spheroids derived from thyroid cancer and contralateral normal tissues are morphologically different

**Figure 1:**
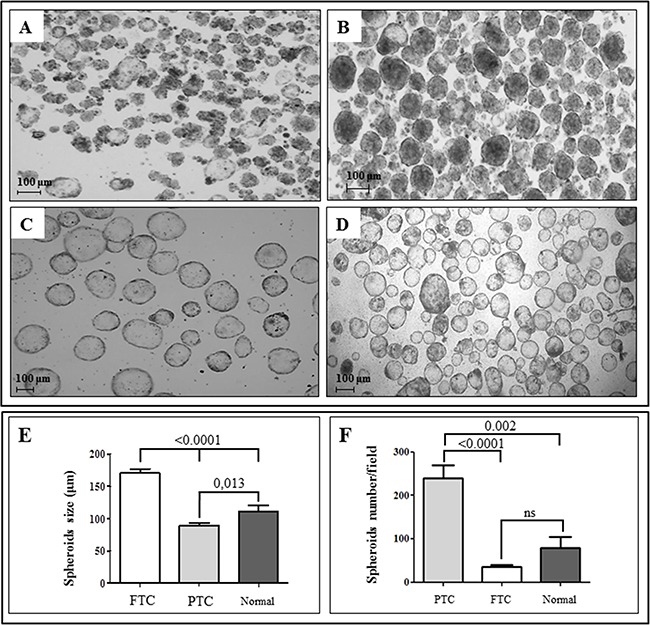
Thyroid spheroids derived from thyroid cancer and contralateral normal tissues are morphologically and numerically different Spheroids display a different morphology depending on the tissue from which they have been obtained. Spheroids obtained from fresh papillary thyroid cancer after 7 days of culture in serum-free and anchorage-independent conditions may display an irregular **A**. or regular **B**. shape. On the other hand, those obtained from follicular thyroid cancer **C**. or normal thyroid tissue **D**. always show, under the same conditions, a regular and more spherical shape. Spheroids from follicular tumors and from normal thyroid tissues generally display a hollow morphology, while spheroids from papillary tumors show a more solid pattern (Original magnification, 50X). Moreover, spheroids derived from follicular thyroid cancers (FTC) are usually larger than those from papillary tumors (PTC) and normal thyroid tissues **E**. while the number of spheroids/well is significantly higher for those derived from PTC with respect to those derived from FTC or normal tissues **F**. Values are given as mean ± SEM from different preparations from independent patients. Spheroids size was calculated using AxioVision software rel. 4.8, while spheroids/well were counted using a Leica DMD108 digital microimaging instrument, considering three fields for each preparation.

After 7 days of culture in low attachment condition and serum-free medium, spheroids were obtained from all 21 thyroid cancers and matched normal specimens. In all cases, the thyroid spheroids were morphologically different according to the tissue of origin. In particular, spheroids obtained from 17 papillary thyroid cancers (PTCs) showed a variable morphology (from irregular to regular shape and border) and size (indicating heterogeneous proliferation rates), whereas spheroids from 4 follicular thyroid cancers (FTCs) and contralateral normal thyroid tissues had a more regular shape and well-defined borders. The number of spheroids/well was significantly higher for those derived from PTC tissues with respect to those derived from FTC or normal tissues (239.2 ± 29.88 *vs* 35.71 ± 4.52 and 78.83 ± 25.41, respectively; P<0.0001 and P=0.002, respectively). Moreover, the size of FTC spheroids was significantly larger than that of PTC and normal spheroids (170.7 ± 6.256 *vs* 89.36 ± 3.877 and 111.2 ± 9.377, respectively; P<0.0001). Finally, PTC spheroids tended to have a more solid appearance with respect to the hollow morphology observed in thyroid spheroids from FTC and normal samples (Figure 1).

### Thyroid tissues and the derived spheroids have the same genetic pattern

Among the 17 PTCs analyzed, 9 harbored the BRAF^V600E^ mutation and 2 a -124 C > T (C228T) TERT mutation, while N-RAS mutations were found in 2 FTCs (Q61R and Q61K) and the C228T TERT mutation in 2 FTCs. In 8 tumors no mutations were identified, though it should be highlight that Sanger sequencing harbors a relatively low sensitivity and there is a possibility that some mutations could have been missed if present in a small percentage of tumor cells. Interestingly, the genetic pattern of the tumor tissues and that of the deriving spheroids was always concordant. In order to test possible variations in the genetic pattern during culture, 3 cases were studied at different time points (7, 14 and 21 days), and no variation in the genetic pattern was observed (Table 1). Moreover, no morphological differences were noted among thyroid tumor spheroids harboring different genetic alterations.

**Table 1:** Clinical and histological characteristics of the tumors included in the study. The genetic characterization is also reported. In all cases the genetic pattern of the tissues and of the matched spheroids are concordant

#	gender	age	histology	pTNM	TERT	BRAF	N-, H-, K-RAS	RET/PTC	TRK/T
1	F	39	CPTC	T1amNX	WT	WT	WT	WT	*nd*
2	F	42	CPTC	T1bN0	WT	V600E	WT	WT	WT
3	F	41	FTC	T2Nx	C228T	*nd*	WT	*nd*	*nd*
5	F	26	CPTC	T3mN1a	WT	V600E	WT	WT	*nd*
7	F	46	CPTC	T1mN0	WT	WT	WT	WT	WT
8	M	32	FVPTC	T3mN1b	WT	WT	WT	WT	*nd*
9*	M	65	CPTC	T3N1b	WT	V600E	WT	WT	WT
10	M	52	CPTC	T3N1a	WT	V600E	WT	WT	WT
11*	F	81	FTC	T3aN0	WT	*nd*	WT	*nd*	*nd*
12	F	44	CPTC	T3mN0	C228T	WT	WT	WT	WT
14	M	28	CPTC	T3N1	WT	WT	WT	WT	WT
15	F	39	FTC + insular	T3mNx	WT	*nd*	Q61R (N)	*nd*	*nd*
19	F	36	CPTC	T3N1a	WT	V600E	WT	WT	*nd*
20*	F	65	FVPTC	T2mN0	WT	V600E	WT	WT	WT
*28*	*F*	*69*	*CPTC*	*T3mN1*	*C228T*	*V600E*	*WT*	*WT*	*WT*
*29*	*F*	*28*	*CPTC*	*T1aN1b*	*WT*	*WT*	*WT*	*WT*	*WT*
*30*	*F*	*35*	*CPTC*	*T3N1a*	*WT*	*WT*	*nd*	*WT*	*nd*
*31*	*F*	*39*	*CPTC*	*T2N1a*	*WT*	*V600E*	*WT*	*WT*	*WT*
*32*	*M*	*35*	*CPTC*	*T2N1a*	*WT*	*WT*	*WT*	*WT*	*WT*
*33*	*F*	*88*	*CPTC*	*T3pN0*	*WT*	*V600E*	*WT*	*WT*	*WT*
*34*	*M*	*67*	*FTC*	*T4bNX*	*C228T*	*nd*	*Q61K (N)*	*nd*	*nd*

*Legend:* CPTC: classical variant papillary thyroid cancer; FVPTC: follicular variant papillary thyroid cancer; FTC: follicular thyroid cancer; WT: wild-type; *nd:* not done. In italics cases submitted to SP treatment. * genetic pattern confirmed in spheroids at 7, 14 and 21 days of culture.

### The expression of thyroid differentiation cell markers is maintained in spheroids derived from PTCs, FTCs and normal thyroid tissues

**Figure 2:**
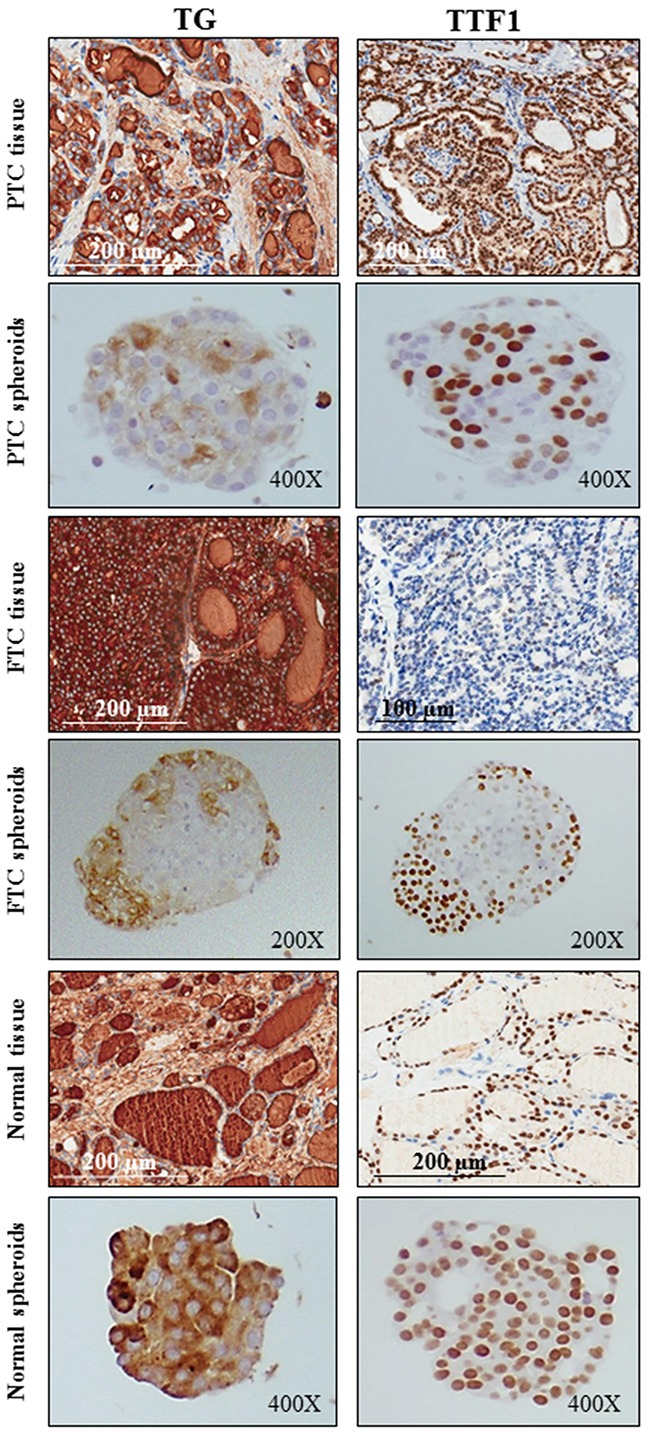
The expression of thyroid differentiation cell markers is maintained in spheroids derived from PTCs, FTCs and normal thyroid tissues The expression of the differentiation markers thyroglobulin (TG) and TTF1 is maintained in both papillary (PTC), follicular (FTC) and normal spheroids (cytoplasm and nucleus, respectively). The expression is variable, mostly sparse in spheroids from normal tissues or preferentially located in the peripheral area of tumor spheroids.

The expression of thyroid differentiation markers, TG (thyroglobulin) and TTF1, was maintained in spheroids and was variable, mostly sparse in spheroids from normal tissues or preferentially located in the peripheral area of tumor spheroids (Figure 2).

### Endothelial and hematopoietic markers are expressed in PTCs, FTCs and normal thyroid tissues and in the derived spheroids

**Figure 3:**
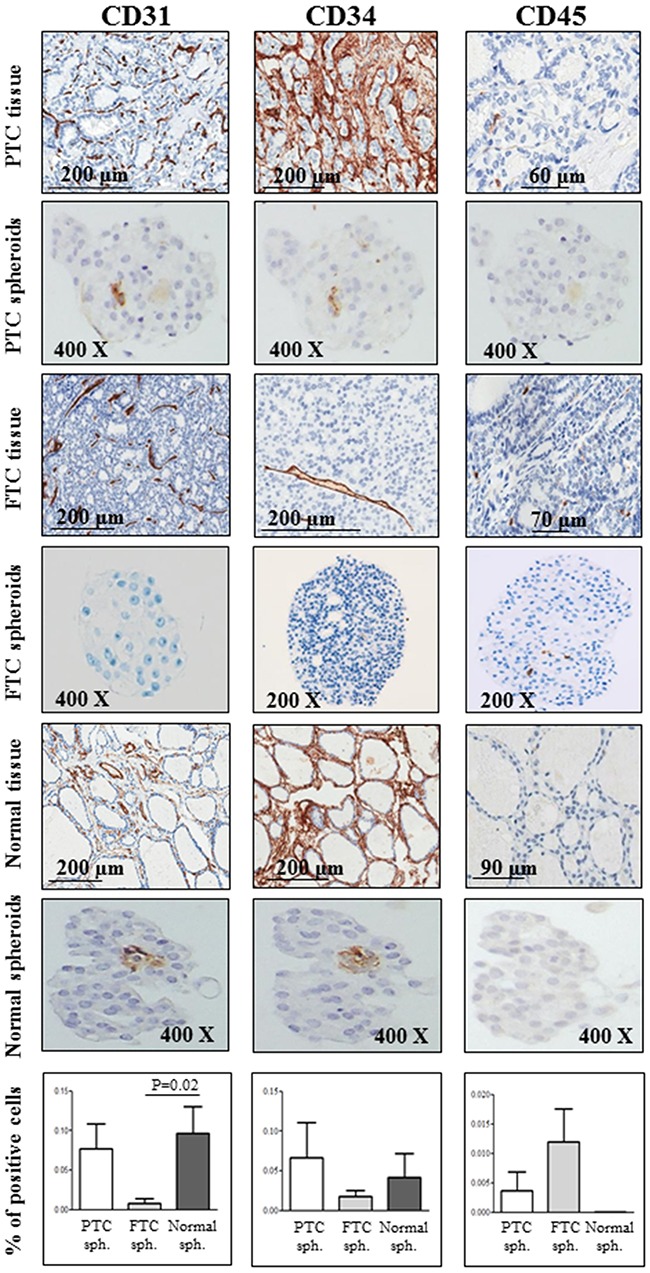
Endothelial and hematopoietic markers are expressed in PTCs, FTCs and normal thyroid tissues and in the derived spheroids A strong staining for the hematopoietic/endothelial marker CD34 and the endothelial antigen CD31 is observed in both papillary thyroid cancer (PTC) and normal spheroids. Interestingly, these two markers localize in the core of the spheroid. A mild or absent expression of both antigens is observed in follicular thyroid cancer (FTC) spheroids. On the contrary, the staining for the leucocyte common antigen CD45 is negative in both PTC and normal thyroid spheroids, and positive in some spheroids from FTC tissues. For CD31, CD34 and CD45, positive cells were scored out of the total cell number within at least 5 spheres for each sample (PTC, FTC and normal spheroids) and expressed as a percentage. Bars represent mean percentages ± SEM. Only significant differences are reported.

The leucocyte common antigen CD45 was never expressed in PTC or normal thyroid spheroids here analysed, while its expression was detected in some cells within FTC spheroids. CD45-positive cells were more present in FTC than in PTC or in normal spheroids. On the other hand, a strong staining for the hematopoietic/endothelial marker CD34 and the endothelial antigen CD31 was found in both PTC and normal spheroids. Interestingly, these positive cells were found to be located in the spheroids’ core, showing a flattened nucleus and a morphology resembling endothelial cells. The mean values of stained cells obtained in multiple replicates of PTC, FTC and normal spheroids are reported in Figure 3.

### Embryonic stem cell markers are expressed in PTCs, FTCs and in the derived spheroids

**Figure 4:**
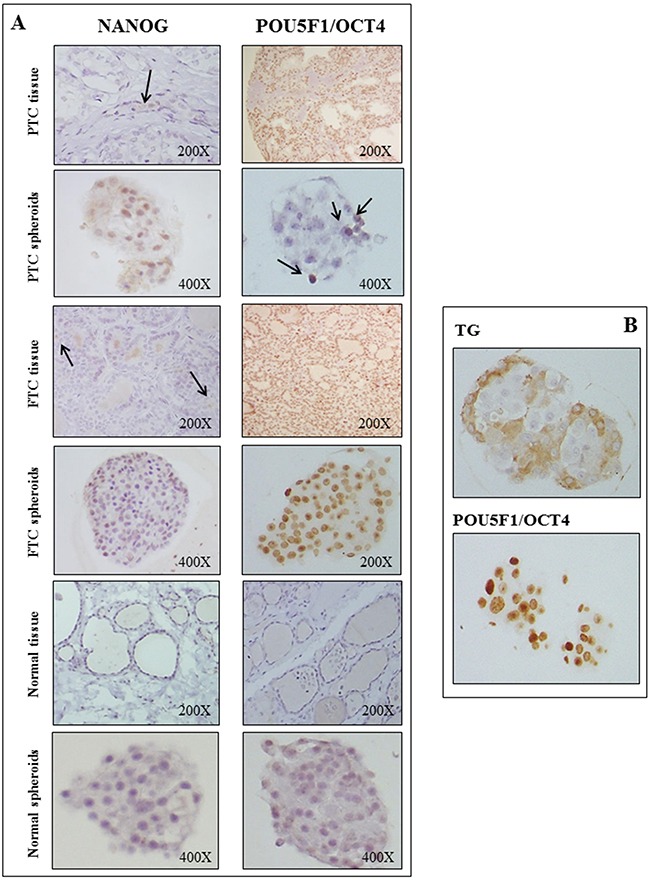
Embryonic stem cell markers are expressed in PTCs, FTCs and in the derived spheroids **Panel A:** In PTC and FTC tissues NANOG is expressed in few cells, whereas a diffuse expression is observed in the corresponding spheroids. POU5F1/OCT4 is expressed in the majority (50-80%) of either PTCs or FTCs tissues, and its expression is maintained also in the corresponding spheroids. In normal tissues the expression of both markers is negative, while it is weak and diffuse in the corresponding normal thyroid spheroids. **Panel B:** The analysis for the stem cell marker POU5F1/OCT4 and the thyroid differentiation marker thyroglobulin (TG) on consecutive paraffin-embedded tumor spheroids sections shows that POU5F1/OCT4 cells mostly locate in the centre and TG cells at the periphery of the spheroids.

Among the nuclear embryonic stem cell markers examined in PTC and FTC sections, NANOG resulted to be expressed in few cells, whereas a diffuse expression was observed in the corresponding spheroids (Figure 4, panel A). At variance, POU5F1/OCT4 was expressed in the majority (50-80%) of either PTCs or FTCs tissues, and its expression was maintained also in the corresponding spheroids (Figure 4, panel A). A weaker expression of these markers was found in both the normal tissues and in the derived spheroids. Interestingly, the immunohistochemistry analysis for the stem cell marker POU5F1/OCT4 and the thyroid differentiation marker TG on consecutive paraffin-embedded tumor spheroids sections allowed us to demonstrate that POU5F1/OCT4 cells mostly located in the centre while TG cells at the periphery of the spheroids (Figure 4, Panel B).

### SP inhibits cell proliferation in multicellular tumor spheroids

**Figure 5:**
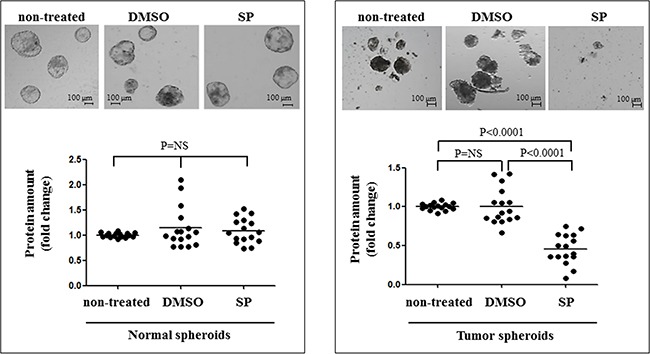
SP inhibits cell proliferation in multicellular tumor spheroids In the upper panels normal and tumor spheroids are shown, either non-treated or DMSO treated (DMSO), or SP600125 (20 μM 96 hrs) treated (SP). The lower panel reports data on the cell proliferation in non-treated and treated normal and tumor spheroids. Tumor spheroids have a significant reduction in the number, the size and the total protein amount after SP treatment.

SP anti-proliferative effects were assessed *in vitro* on thyroid multicellular spheroids derived from tumors and their corresponding normal thyroid tissues, carrying different genetic alterations (patients #28-34). SP was highly effective against both PTC and FTC spheroids, whereas no effect was observed in non-treated or in DMSO treated tumor spheroids. In particular, tumor spheroids surviving 96 hours of SP treatment appeared as smaller, inhomogeneous and irregular aggregates. This was consistent with the significant reduction in the protein content with respect to untreated spheroids (P<0.0001, Figure 5). Importantly, no effects were noted in normal spheroids. It is worth to note that SP anti-proliferative effects were not different among PTC and FTC cases, and among mutated or non-mutated spheroids (data not shown), though the sample analyzed is too limited to draw definite conclusions.

### SP exerts its main effects through direct inhibition of ROCK pathway

**Figure 6:**
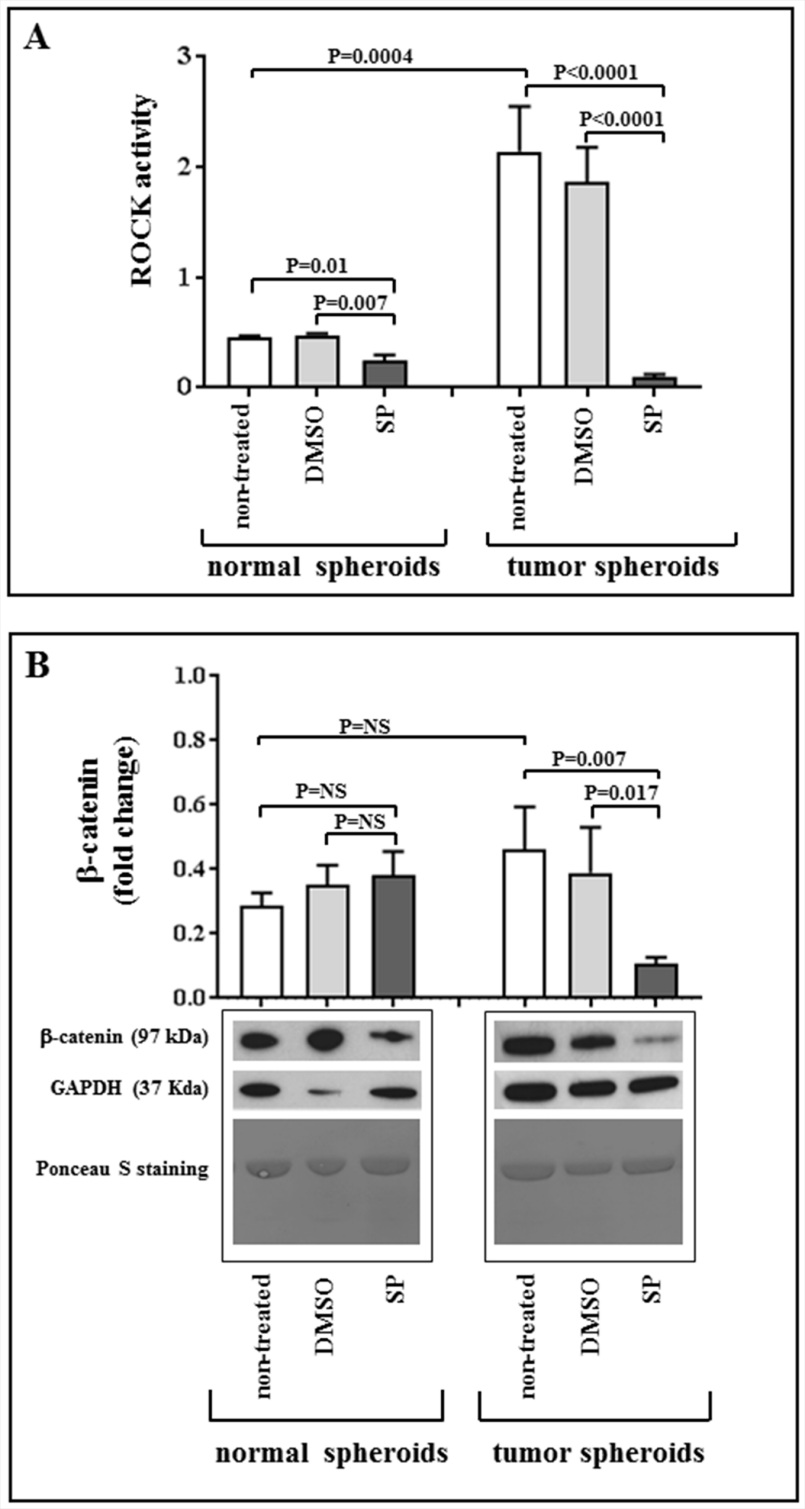
SP acts through ROCK pathway inhibition **Panel A:** Basal status of ROCK pathway and SP-induced modifications in normal and tumor spheroids. Quantification of ROCK activity at baseline and after incubation with 20 μM SP or equivalent amount of DMSO for 96 hours. Values are given as mean ± SEM. **Panel B:** Quantification of β-catenin expression and representative images of western blots showing total amount of β-catenin in non-treated spheroids, and after incubation of normal and tumor spheroids with 20 μM SP or equivalent amount of DMSO for 96 hours; GAPDH was used as loading control. Ponceau S staining on the same membranes is also reported, confirming the uniformity of protein total amount in each sample. No significant variation in sample quantification is detected when normalizing for GAPDH or Ponceau staining. Values are given as mean ± SEM.

The tumor spheroids had a significantly higher ROCK activity than normal spheroids (P=0.0004, Figure 6 panel A). SP treatment was found to significantly reduce the ROCK activity in tumors spheroids and, at a lesser extent, in normal spheroids (P<0.0001 and P=0.007, Figure 6 panel A). As a second step, we examined whether these results were concordant with changes in the downstream pathway, and in particular in the β-catenin levels. Consistent with ROCK activity data, tumor spheroids showed a significant decrease in the β-catenin expression after SP treatment, whereas no differences were observed between treated and untreated normal spheroids (Figure 6, panel B).

## DISCUSSION

Drug attrition rates for cancer are much higher than in other therapeutic areas. Only 5% of agents that have anticancer activity in preclinical development demonstrates a sufficient efficacy in phase III testing and reaches the patients [18]. This is not only an unacceptable waste of money, but means the loss of opportunities for those patients participating in unsuccessful clinical trials. The high attrition rates are largely due to the inappropriate conditions used in the preclinical phases that are poorly representative of the biological characteristics of the original tumor [19]. In this context, multicellular spheroids are emerged as an experimental model intermediate in their complexity between monolayer cultures and patients’ tumor [1, 2]. Several methods have been developed to generate multicellular tumor spheroids, some of which are scaffold-based (e.g., matrix-on-top, matrix-embedded, matrix encapsulation, spinner flasks, micropatterned plates, liquid overlay) while others are scaffold-free methods (e.g., ultra-low attachment plates, hanging drop, rotary cell culture systems). In scaffold-based methods, extracellular membrane-based natural hydrogels (such as Matrigel or Agar) and synthetic or engineered hydrogels are commonly used. Among scaffold-free methods, promising NASA rotary cell culture systems (Random Positioning Machine and Rotating wall vessels), which simulate a near-weightlessness environment or microgravity are included [2, 20].

In the present study, a model of multicellular tumor and normal thyroid spheroids was established using nonadherent conditions (ultra-low attachment plates). We show that spheroids are morphologically and numerically different according to the tissue of origin and have a genetic pattern always concordant with that of the corresponding tissue. The TG and TTF1 thyroid differentiation markers are expressed both in tumor and normal spheroids. The expression is lower in tumor spheroids, and very weak in those derived from PTCs, compared to normal spheroids.

In thyroid tumor tissues, contrasting and extremely partial data on stem markers expression are available in the literature. Indeed, in differentiated and undifferentiated thyroid cancers, POU5F1/OCT4 was variably expressed in some cases [21, 22], but not in others [23, 24]. In the tissues here characterized, POU5F1/OCT4 is the stem cell marker with the highest expression, with a particularly strong staining in FTCs. The expression of this stem gene is absent in normal thyroid tissues contralateral to tumors, consistent with recently reported data [16]. The immunostaining of tumor spheroids confirms the findings at the tissue level, with a strong expression of POU5F1/OCT4, particularly in FTC spheroids. Interestingly, by means of immunohistochemistry on consecutive slides, we show that the stem marker POU5F1/OCT4 is mainly expressed in the core of spheroids, whereas TG has a peripheral expression. This is consistent with the notion that stem-like cells preferentially reside in niches characterized by hypoxia and low nutrients, such as the core of the spheroids.

Interestingly, in the spheroids core we show the expression of the hematopoietic/endothelial marker CD34 and the endothelial antigen CD31 in cells, displaying a flattened nucleus typical of endothelial cells and mimicking the formation of vascular structures. However, the expression of hematopoietic and endothelial markers was heterogeneous among the different tumors and the corresponding spheroids.

As a second step, we evaluated the performance of this spheroid model in the testing of new molecules of potential interest in anticancer treatment. Currently, several tyrosine kinase inhibitors (such as Sorafenib or Lenvatinib) are used in protocols or as approved drugs in the treatment of advanced radioiodine-refractory thyroid cancer [25]. Nevertheless, the actual improvement of overall survival is questioned, the side effects have a major impact on the quality of life, and the resistance to treatment ensues with time in virtually all treated patients, likely due to the development of compensatory pathways and to the persistence of cancer stem cells. Thus, there is still the need to generate new and more effective drugs. We recently demonstrated that a small compound, SP600125 (SP), is able to induce the inhibition of cancer cell migration and cell death through mitotic catastrophe [8]. The effect was shown in two-dimensional cell cultures of well-differentiated, poorly differentiated and anaplastic thyroid cancer, being the highest effects observed in poorly differentiated and anaplastic cell lines [8]. In this study, we report the potent effect of SP on cell survival in our 3D multicellular cultures of neoplastic thyroid spheroids derived from papillary and follicular thyroid cancers. In particular, tumor spheroids surviving 96 hours of SP treatment become smaller, inhomogeneous and irregular, with a significant reduction in the protein content with respect to untreated spheroids. Importantly, the effect is selective on tumor spheroids in comparison to normal thyroid spheroids.

In cancer tissues and monolayer cultures [8], we found an increased ROCK activity and SP was shown to exert its main effect through direct and highly significant inhibition of this kinase that is involved in the regulation of cell migration, microtubule dynamics and β-catenin turnover [26, 27]. Indeed, β-catenin can act as pro-proliferative factor and is involved in cancer invasiveness and metastatization, and its activity was found to be increased in several undifferentiated cancers [28, 29]. Here we show that ROCK activity is significantly higher in neoplastic spheroids than in normal ones, and SP treatment induces a significant decrease of ROCK activity and β-catenin levels in cancer spheroids, while minor or no effects were recorded in spheroids from normal tissues. These data further support SP as a promising therapeutic compound for thyroid cancer.

ROCK inhibitors are currently used in clinical trials for glaucoma, Raynaud phenomenon or vasoconstriction-related diseases, and erectile dysfunction, either as ophthalmic solutions or as preparations for oral administration (https://clinicaltrials.gov/; https://www.clinicaltrialsregister.eu/). For either the treatment of these diseases or as anticancer drugs, ROCK inhibitors have been tested at concentrations ranging 10-100 μM for *in vitro*, and 1-200 mg/kg/day for *in vivo* studies [30, 31]. According to the data available so far, SP600125 appears to be as effective as currently tested ROCK inhibitors, or even more effective. Indeed, we confirmed its efficacy at 20 μM concentration in accord with previous data reporting that in cell cultures it acts at concentrations as low as 5-20 μM, and in mice it reduces p53-defective cancer cells growth at the daily dose of 2.5 mg/kg [32].

In conclusion, we report a morphological and molecular characterization of normal and tumor thyroid spheroids. The analysis of the corresponding tissues, allows to conclude that the spheroids well recapitulate the characteristics of the tissues of origin, either in the differentiated and “stem-like” components. Another significant advantage of the spheroid model is the possibility to test cells derived from the normal tissues. This 3D model allowed to test *in vitro* the effects of the kinase inhibitor SP, which was found to be highly effective on both PTC and FTC derived spheroids. The effect of SP appears to be selective on tumor spheroids and it involves the direct inhibition of the ROCK-β-catenin pathway. This mechanism of action may be particularly important in anticancer therapy considering that Rho/ROCK/ β-catenin pathway is hyperactivated in neoplasia, and its activity correlates with metastatic disease [8–11]. Further studies will be devoted to test other novel molecules in this spheroid model in order to identify possible novel drugs selective for neoplastic cells, including those with “stem-like” features.

## MATERIALS AND METHODS

### Chemicals

Cell culture reagents, Restore Western Blot Stripping reagent, monoclonal anti-GAPDH antibody and HRP conjugated secondary antibodies were purchased from Thermo Fisher Scientific (Waltham, Massachusetts, USA). SP600125 (1,9-Pyrazoloanthrone), DMSO, Ponceau S, collagenase type I, monoclonal anti-β-Catenin and anti-α-Tubulin antibodies were purchased from Sigma-Aldrich (Saint Louis, MO, USA). Fibroblast growth factor (bFGF) and epidermal growth factor (EGF) were purchased from PeproTech EC (London, UK). Monoclonal anti-POU5F1/OCT4 antibody was purchased from Santa Cruz Biotechnology Inc. (Dallas, TX, USA); polyclonal anti-NANOG antibody from ReproCELL Inc. (Yokohama, Kanagawa, Japan), monoclonal anti-TTF1, TG, CD31, CD34 and CD45 antibodies from Cell Marque (Rocklin, CA, USA), and monoclonal anti-Actin Ab-5 from BD Italia, (Milan, Italy). Protease inhibitors cocktail tablets were purchased from Roche (Basel, Switzerland).

### Samples

A total of 17 papillary (PTC) and 4 follicular (FTC) tumor tissues, and the corresponding contralateral normal tissues were obtained from patients undergoing thyroidectomy for thyroid cancer (Table 1). All samples were obtained at the time of surgery in the Endocrine Surgery Unit, and immediately dissected by the pathologist under sterile conditions. In particular, the “core” of the tumor was carefully isolated to ensure the highest percentage of tumor cells and to avoid contamination with normal thyroid tissue. Fresh specimens were used for spheroids cultures and genetic analyses, while the corresponding paraffin embedded samples were used for immunohistochemistry analysis. All patients gave informed consent to the study, which was approved by the local Ethical Committee.

### Thyroid spheroids cultures

Tumor and normal thyroid tissues were digested in DMEM/2 mg/ml collagenase type I (Sigma-Aldrich, Saint Louis, MO, USA) at 37°C for 2 h. Cells were seeded at 5×10^5^ cells and grown in serum-free medium: DMEM/F12 1:1 medium (Gibco-Thermo Fisher Scientific, Waltham, Massachusetts, USA) containing 20 ng/ml human basic fibroblast growth factor-bFGF (PeproTech EC, London, UK), 20 ng/ml human epidermal growth factor-EGF (PeproTech EC, London, UK), B27 (1:50 dilution, Gibco-Invitrogen, Carlsbad, CA, USA), antibiotics and Fungizone (Gibco-Thermo Fisher Scientific Waltham, Massachusetts, USA) in ultra-low attachment 6 well plates (Corning Incorporated Life Science, Tewksbury, MA, USA). The wells of the ultra-low attachment plates are coated with polystyrene, an inert substrate, which blocks cell attachment and induces cells in suspension to aggregate into visible spheroids. The cells were incubated in a 37°C, 5% CO_2_ incubator and floating primary aggregates of cells with spheroid-like structure appeared after 7 days, and fresh medium was added to wells weekly. Spheroids/well were counted using a Leica DMD108 digital microimaging instrument (Leica Microsystems, Milan, Italy) and the spheroids diameter was calculated using AxioVision software rel. 4.8 (Oberkochen, Germany).

### Nucleic acids isolation and genetic characterization of matched tissues and thyroid spheroids

All tissues and thyroid spheroids were submitted to genetic characterization. According to data available in the literature related to the most common mutational pattern of different thyroid cancer histotypes, we screened PTCs for RET and TRK common rearrangements, TERT, BRAF and RAS common point mutations, and FTCs only for TERT and RAS mutations [33–36]. The analysis of TRK and/or RAS was not done in 5 (#1, 5, 8, 19, 30) papillary tumors, due to the lack of high quality material. Nucleic acids were extracted from tissues samples, by means of a commercial kit (Puregene® Core kit A, Qiagen Sciences, Maryland, USA) and from thyroid spheroids using the AllPrep DNA/RNA Micro Kit (Qiagen GmbH, Hilden, Germany) according to the manufacturer's specifications. DNAs were then used for the screening of known point mutations in the BRAF, H-, N-, K-RAS, TERT genes, while RNAs were retro-transcribed and amplified with appropriate primers for the identification of TRK and TRK-T1/T2/T3 and RET-PTC1/PTC2/PTC3 rearrangements, as previously described [33, 37–41]. Primers and PCR conditions used are reported in the Supplementary Table 1.

### Immunostaining of thyroid spheroids and tissues

Thyroid spheroids were harvested after 7 days, centrifuged at 300g for 5 minutes and the pellet was included in 1% Agar in PBS, fixed in formalin and paraffin-embedded. Consecutive paraffin sections of 3 μm were cut and used for immunohistochemistry analyses. The first slide was stained with hematoxylin/eosin for morphological examination of the spheroids. Subsequent slides were submitted to immunostaining with antibodies against markers of stem cells (POU5F1/OCT4, Santa Cruz Biotechnology Inc., Dallas, TX, USA; NANOG, ReproCELL Inc., Yokohama, Kanagawa, Japan), thyroid progenitors (TTF1, Cell Marque, Rocklin, CA, USA), mature thyroid cells (TG, Cell Marque, Rocklin, CA, USA), endothelial cells (CD31, Cell Marque, Rocklin, CA, USA), hematopoietic progenitor/stem cells (CD34, Cell Marque, Rocklin, CA, USA), leucocytes (CD45, Cell Marque, Rocklin, CA, USA). Clones, commercial brands, dilutions and length of incubation/temperature of the antibodies used are reported in Supplementary Table 2. Appropriate secondary antibodies and detection systems were used in an automatic immunostainer (Bench Mark Ultra, Ventana Medical Systems Inc., Tucson, USA). Staining was revealed using the UltraView Universal DAB (Ventana Medical Systems Inc., Tucson, USA) and counterstained with hematoxylin. Paraffin sections of appropriate tissues (tonsil for CD31, CD34 and CD45; thyroid for thyroglobulin; lung adenocarcinoma for TTF1; seminoma for POU5F1/OCT4 and NANOG) were used as positive controls. Primary antibody omission was used as negative control. For quantification of immunoreactivity, four high-power fields per tissue and at least 10 spheroids per case were viewed by two independent pathologists (SB and SF). Representative images of all stainings were then obtained using a Leica DMD108 digital microimaging instrument (Leica Microsystems, Milan, Italy). For CD31, CD34 and CD45 positive cells were scored out of the total number on cells within at least 5 spheres for each sample and expressed as a percentage. The staining intensity was not considered due to homogeneous expression of the markers among samples.

### SP600125 (SP) treatment

SP anti-proliferative effects were assessed on thyroid multicellular spheroids derived from 6 PTCs, 1 FTC and their corresponding normal spheroids. Tumor and normal thyroid spheroids were obtained, as described above, plating cells at a density of 10^5^ cells per well in ultra-low attachment 24-well plates (Corning Incorporated Life Science, Tewksbury, MA, USA). SP600125 (Sigma-Aldrich, Saint Louis, MO, USA) was dissolved in DMSO (Sigma-Aldrich, Saint Louis, MO, USA) to yield a stock solution of 20 mM, which was stored at −20°C. SP concentration to be used (20 μM) and the duration of treatment (96 hours), were chosen based on our previous data on monolayer cultures [8] and on preliminary experiments (not shown). Non-treated thyroid spheroids were also used as control. The plates were then placed in a 37°C, 5 % CO_2_ incubator. After 4 days of treatment, cells were harvested for proteins extraction. Spheroids/well were manually counted using a Leica DMD108 digital microimaging instrument (Leica Microsystems, Milan, Italy) and the scale bars were applied using AxioVision software rel. 4.8 (Oberkochen, Germany).

### Protein extraction and proliferation assay

Briefly, the pellet of the spheroids obtained from 6 PTCs, 1 FTC and from their corresponding normal thyroid tissues were grown for 2 weeks and harvested for non-treated, DMSO treated (DMSO, 20 μM) and SP treated (SP, 20 μM) evaluation after additional 96 hours. They were homogenized and lysed in a constant amount of RIPA buffer (10 mM Tris-HCl pH 7.5, 500 mM NaCl, 0.1% SDS, 1% NP40, 1% Na-deoxycholate, 2 mM EDTA), supplemented with complete protease inhibitors cocktail tablets (Roche, Basel, Switzerland). After sonication, 5μL of protein extracts were used for protein determination with Pierce BCA protein assay kit (Thermo Fisher, Waltham, Massachusetts, USA). Each sample was measured in duplicate and samples immediately stored at -80°C.

### Western blot analysis

Thirty micrograms of protein extracts were separated on NuPAGE Novex 4-12% Bis-Tris Protein Gels (Thermo Fisher Scientific, Waltham, Massachusetts, USA) and electrotransferred to nitrocellulose membranes with iBlot System (Thermo Fisher Scientific, Waltham, Massachusetts, USA). The quality and uniformity of protein transfer was checked through Ponceau S solution staining (Sigma-Aldrich, Saint Louis, MO, USA). Membranes were blocked with 5% milk TBS-T, probed overnight at 4°C with β-catenin primary antibody and incubated with the appropriate HRP-conjugated secondary antibody for 1 h at room temperature. GAPDH (Ambion-Thermo Fisher Scientific, Waltham, Massachusetts, USA) was used as housekeeping control. In preliminary experiments, samples were blotted with different proteins (actin, α-tubulin and GAPDH) usually used as loading control, and GAPDH was found to be the most reliable. The results obtained with GAPDH were comparable to those obtained with Ponceau S staining, which is often used as an alternative method to evaluate the total protein amount [42]. The detection was performed using the Luminata Forte Western HRP substrate (Merck Millipore, Vimodrone, Italy). Bands of interest were quantified using ImageJ software version 1.47 (National Institute of Health, Bethesda, MD). In order to reduce interindividual variability, tissue extracts were used as reference control. Each sample set (normal or tumor tissue derived) was then normalized accordingly. Clones, commercial brands, dilutions and length of incubation/temperature of all the antibodies used are reported in Supplementary Table 2.

### ROCK activity assay

ROCK activity was measured with Rho-associated Kinase (ROCK) Activity Assay (Millipore, Billerica, MA, USA). Five micrograms of protein extracts were loaded per well and enzyme activity was subsequently determined following the manufacturer's instructions. Each sample was measured in duplicate. Colorimetric reaction was detected at 450nm using ELx800 Absorbance Microplate Reader (BioTek, Winooski, VT, USA).

### Statistical analysis

Statistical analyses were performed with GraphPad Prism Software, Inc., version 5.04 (La Jolla, CA USA). The normality of samples distribution was checked with Kolmogorov-Smirnov test. Given that some of the samples resulted not normally distributed within each group, and also considering the low number of patients tested with SP treatment, the normality assumptions were considered as violated. We thus checked if our data sets were statistically different from each other with non-parametric Mann-Whitney test for direct comparison of two sample sets, and with non-parametric ANOVA on ranks when more than two sample sets were compared. Differences among the sizes and the number of spheroids derived from different tissues and among the percentages of staining for endothelial and hematopoietic markers were evaluated by t-test. All experiments were performed at least four times in duplicate. The difference between values was considered significant when P<0.05.

## SUPPLEMENTARY TABLES



## Abbreviations

3D: three-dimensional
PTC: papillary thyroid cancer
FTC: follicular thyroid cancer
SP: SP600125
TC: thyroid cancer
CSCs: cancer stem cells
TG: thyroglobulin
DMSO: Dimethyl sulfoxide
HRP: horseradish peroxidase
bFGF: basic fibroblast growth factor
EGF: epidermal growth factor
DMEM: Dulbecco's Modified Eagle's medium
PBS: Phosphate-buffered saline
DAB: 3,3′-Diaminobenzidine
Tris-HCl: Tris hydrochloride
NaCl: sodium chloride
SDS: sodium dodecyl sulfate
NP40: nonyl phenoxypolyethoxylethanol
EDTA: Ethylenediaminetetraacetic acid
BCA: bicinchoninic acid
TBS-T: Tris Buffered Saline with Tween 20
SEM: standard error of the mean
hrs: hours
RT: room temperature
o/n: overnight
CPTC: classical variant papillary thyroid cancer
FVPTC: follicular variant papillary thyroid cancer
WT: wild-type.
