# Supplementary material for: Multicellular spheroids from normal and neoplastic thyroid tissues as a suitable model to test the effects of multikinase inhibitors
Source: Oncotarget. 2016 Dec 26;8(6):9752–66. doi: 10.18632/oncotarget.14187 (PMC5354768; doi:10.18632/oncotarget.14187)
Supplement: Supplementary file 1 [file oncotarget-08-9752-s001.pdf]

Multicellular spheroids from normal and neoplastic thyroid tissues as a suitable model to test the effects of multikinase inhibitors

SUPPLEMENTARY TABLES

Supplementary Table 1: List of the primers used for the amplification and the sequencing of *BRAF*, *TERT, H-, N-, K-RAS* point mutations and *RET/PTC* and *TRK* rearrangements

Primers	Sequence (5 to 3’)	Annealing	References
BRAF(exon 15)			
F	TCATAATGCTTGCTCTGATAGGA	60°C	Muzza M *et al.*, *Mol Cell Endocrinol*. 2015
R	GGCCAAAATTTAATCAGTGGA		
TERT promoter			
F	AGTGGATTCGCGGGCACAGA	58°C	Muzza M *et al.*, *Mol Cell Endocrinol*. 2015
R	GCAGCGCTGCCTGAAACTC		
H-RAS (exons 1-2)			
1F	CAGGAGACCCTGTAGGAGGA	62°C	Moura MM *et al.*, *J Clin Endocrinol Metab*. 2011
1R	CCTATCCTGGCTGTGTCCTG		
2F	TCCCTGAGCCCTGTCCTC	62°C	
2R	CAGCCTCACGGGGTTCAC		
N-RAS (exons 2-3)			
2F	CCAAATGGAAGGTCACACTAG	58°C	Newly drawn
2R	TGATCCGACAAGTGAGAGACA		
3F	ACCTTGGCAATAGCATTGCAT	55°C	Newly drawn
3R	TAGTGTGGTAACCTCATTTCC		
K-RAS (exon 3)			
3F	CTTTGGAGCAGGAACAATGTC	58,4°C	Newly drawn
3R	AACAGGGATATTACCTACCTC		
RET/PTC1			
F	ATTGTCTCTCGCCGTTC	60°C	Muzza M *et al.*, *Clin Endocrinol (Oxf)* 2010
R	CCTGCTCTGCCTTTCAGATGGAA		
RET/PTC2			
F	GTGATTGATCAAGGAGAGACG	57°C	Muzza M *et al.*, *Clin Endocrinol (Oxf)* 2010
R	AGGGAATTCCCACTTTGGATC		
RET/PTC3			
F	TGGAGAAGAGGAGCTGTATCT	60°C	Muzza M *et al.*, *Clin Endocrinol (Oxf)* 2010
R	AGGGAATTCCCACTTTGGATC		

(*Continued*)

Primers	Sequence (5 to 3')	Annealing	References
TRK-T1			
F	GCTGAGAAAAGAGACTTAAT	55°C	Greco A *et al.*, *Cell Growth Differ.*, 1993
R	CATCACTGAAGTATTGTG		
TRK-T2			
F	TTTGAGGGAGCAAATGAGA	52°C	Greco A *et al.*, *Genes Chrom and Cancer.* 1997
R	CACTTGAGCACGATGTC		
TRK-T3			
F	AACTTCGAAATAAAGTGAAT	50°C	Frattini M *et al.*, *Oncogene* 2010
R	CAAACTTGTTTCTCCGTCCAC		
TRK			
F	GTGTCTGAGTGCTGCCGAAGA	62°C	Frattini M *et al.*, *Oncogene* 2010
R	CAAACTTGTTTCTCCGTCCAC		

Supplementary Table 2: List of the primary antibodies used for immunohistochemistry (OCT4, NANOG, CD31, CD34, CD45, TG, TTF1) and for western blot analyses (β-Catenin, GAPDH, Actin and α-tubulin)

Antibody	Clone	Commercial brand	Dilution	Temperature/ incubation length
OCT 3/4	C-10	Santa Cruz Biotechnology Inc.	1:100	RT/1 hour
NANOG	Rabbit polyclonal	ReproCELL	1:100	RT/1 hour
CD31	JC70	Cell Marque	Pure	37°C/16 min
CD34	QBEnd/10	Ventana	Pure	37°C/16 min
CD45	2B11 & PD7/26	Cell Marque	Pure	37°C/20 min
TG	MRQ-41	Cell Marque	Pure	37°C/32 min
TTF-1	8G7G3/1	Ventana	Pure	37°C/32 min
β-Catenin	6F9	Sigma-Aldrich	1:2000	4°C o/n
GAPDH	6C5	Thermo Fisher Scientific	1:4000	4°C o/n
Actin Ab-5	C4/actin	BD Italia	1:4000	4°C o/n
α-Tubulin	DM1A	Sigma-Aldrich	1:4000	4°C o/n

Legend: RT, room temperature; o/n, overnight.
